# Two Cases of Disease in the Circulating System

**Published:** 1831

**Authors:** 


### Periscope;

OR,

### CIRCUMSPECTIVE REVIEW.

"Ore trahit quodcunque potest, atque addit acervo."

I.

### Two Cases of Disease in the Circulating System. By Dr. J. P. Kay, Physician to the Ardwick and Ancoats Dispensary, Manchester.

These cases are related by Dr. Kay, in the third Number of our highly respectable contemporary, the North of England Medical and Surgical Journal.

Case 1. "E. F. an old woman, (aged 75) who had been some time bed-ridden, had paralysis of the muscles, accompanied by great loss of sensation, throughout the whole of the left side of the body, which had occurred two years before the period when I first saw her. She was then evidently suffering from very general organic disease. She was not much more emaciated and withered than might be expected in a feeble woman of her age, but the peculiarity of her physiognomical expression indicated serious lesion of some important organ, besides the disease of which external evidence existed.

When I first saw her, slight spots of gangrene had appeared upon the extremities of the toes of the right foot. Her strength was supported by tonics and stimulants, but these, with the aid of local applications, seemed to exert little influence over the progress of the disease. The gangrenous spots gradually spread, encroached on the surrounding parts, and in the course of a month, by a slow, but uniform progress, had affected one-half of the foot. The soft parts slowly sloughed out, with little or no discharge, and left the bones of the meta-tarsus bare. The foot, above the line of the gangrene, became somewhat œdematous.

The femoral artery in the right groin beat much less forcibly than usual. The leg was drawn up a little, and gradually became more so, until it was bent nearly to a right angle at the knee. This appeared *not* to be accidental, and the limb could not be straightened without much pain and considerable difficulty. There was, however, no muscular rigidity; the muscles had indeed lost almost all power. Accidental motions of the leg, as when it was displaced by the carelessness of an attendant, gave great pain in all the tissues. It was difficult, from the age and weakness of the patient, to discover whether any slight power of voluntary motion remained in the extremity. I found the leg always in the same position, excepting that it gradually became more flexed, and she affirmed that she had no command over it, and could not, by the greatest efforts, move it. Her strength gradually declined, and, at the end of the fifth week, she died. Twenty-eight hours after death, I proceeded to examine the body.

Eleven years before, it appeared, from the history given to us by the attendants, she had fallen, and wounded her head, and an exfoliation of the upper part of the frontal and parietal bones had taken place. The integuments and the pericranium, as well as the bones themselves, were exceedingly thin, in the situation where the shock of the fall had been received. The dura mater adhered very firmly to this portion internally.

The brain was removed. On examining its exterior, I discovered a considerable effusion of serum beneath the arachnoid membrane. About three ozs. also flowed from the base of the cranium, and from the spinal canal. The basilar and vertebral arteries were diseased. Their lining membrane was opaque in many portions, the coats were brittle, the artery distended without increased growth of tissue, and cartilaginous and caseous deposits were found beneath the internal coat. This disease extended to several small arteries in the brain, and was particularly evident in those of the Corpus Callosum. The cerebrum was firm, and exhibited, when divided by the scalpel, many bloody points. The ventricles contained much serum. The left hemisphere was, in other respects, every where healthy. An abscess was discovered in the posterior part of the right corpus striatum, which stretched beneath the tænia to the left thalamus. The surface of the thalamus was softened, broken down, and presented shreds, which floated in the serum contained in the ventricle.

The left lung was very emphysematous: it contained in the superior lobe a cavern formed by the softening of tubercular matter, and exhibited in the lower lobe a bulla of the size of a large apple. The parietes of the right ventricle of the heart were discovered to be extremely thin, little muscular fibre was left. Only a few fasciculi were scattered through adipose substance and cellular membrane. The parietes of the left ventricle were invaded by a similar degeneration, but not to the same extent. The mitral valve was much thickened, and granular deposits of a somewhat tubercular character were scattered over it, and the semilunar valves of the aorta were somewhat enlarged at their base.

Some spots of deposition occurred beneath the lining membrane of the aorta, in its ascending portion, but from the arch onwards it exhibited a continued and unusual state of disease. Plates of bone had been deposited beneath its lining membrane: in some places, they were completely covered by it; but, in others, only partially, from its subsequent ulceration. Occasionally, this ulceration had proceeded so far as to divest the plate of bone of its membranes over three-fourths of its extent, and it then projected into the cavity of the artery, powerfully impeding the progress of the blood. Among these osseous depositions were interspersed cartilaginous layers, and portions which resembled tubercular secretion. There were also some few abscesses, and in two or three instances organized lymph, which, from its colour, appeared to have been occasioned by an extravasation of blood beneath the lining membrane. All the small arteries which communicated with this portion of the aorta were similarly diseased. At the first lumbar vertebra the artery was slightly contracted. Here the osseous deposit surrounded the whole of its cavity and prevented its collapse, even under strong pressure. It appeared a strong bony tube, and preserved this character beyond the bifurcation almost to the internal ring.

The disease was traced into the internal iliac, and bony deposits could be felt in all the small arteries of the pelvis. The arteries which escape from the pelvis by the ischiatic notch were all similarly diseased on both sides. Both the gluteal arteries were curiously obstructed. Their coats were firmly ossified: a clot appeared to have been formed in their cavities, from the loss of action and roughness of the internal membrane, which, presenting obstructions, had accumulated deposits, and at length occasioned a stagnation and clot. Subsequently, this lymph had become organized, and having partaken of the general tendency to osseous deposition, had become a piece of bone filling the cavity of the artery, and adhering to its internal parietes.

The coats of the right femoral artery were much more disorganized than those of the left, but the same disease prevailed in both vessels, and also in the profunda femoris. In the right femoral, however, at the situation where the tendon of the adductor crosses the artery before it enters the popliteal space, the cavity was obliterated, and the artery beneath contained a clot which filled four or five inches of the main trunk and subdivisions, and it was not further traced. The obliteration was occasioned by circumstances similar to those which existed in the aorta. A portion of the thickened lining membrane had been set free by ulceration, or by rupture from some sudden motion of the leg, and, rolling upwards in the cavity of the artery, had at once effectually obstructed it as with a valve, and prevented the progress of the blood. The left femoral was not obstructed.

Some hours had been spent in making this examination, when the lateness of the evening, and some pressing engagements, reluctantly compelled me to desist from prosecuting this interesting inspection into the smaller branches, and into the brachial and carotid arteries and their ramifications. There can be little doubt, that many of the smaller arteries would have exhibited specimens of the peculiar methods of obliteration discovered in the gluteal and femoral.

This remarkable instance of disease in the lining membrane of the arteries seems to indicate the source from which even ossific deposits sometimes arise. Many of the peculiar products discovered in the tissue of the arteries seemed to be the immediate consequences of inflammation. Abscesses and lymph are its common terminations. The osseous and cartilaginous layers may, therefore, be considered as resulting from morbid actions which ensued in the effused lymph after its organization, and which being connected with the tissue of the artery (which is itself, from '*depraved nutrition*' liable to these changes) became the bed of ossific deposits. The peculiar process by which the lining membrane of the artery is separated from the subjacent coats, whether by violence or simply by diseased action, is well described by Mr. Turner,* the Professor of Surgery to the Royal College of Surgeons of Edinburgh; and this case affords an exemplification of the opinions which he has so ably supported. The portions projecting into the interior of the aorta to a certain degree impeded the flow of blood, and threatened to form still greater obstacles.

It becomes of consequence that we should be able to recognize a malady of such importance in its origin, when, as in some of the cases on record, the inflammation is acute, and may be treated in the ordinary way. Subsequently, we can have little hope of arresting the progress of such serious disease existing in a tissue of low vascular organization, and little susceptible, as far as we can judge, of influence from external or internal remedies.

The symptoms in the acute stage must vary as the inflammation is local or general, and even when it affects the general arterial system its history appears to be vague.† After rigors, the pulse becomes quick and hard, and the general symptoms of acute inflammatory action supervene. They are accompanied frequently by slight cough, diffused pain in some portion of the chest or back, and sometimes by continued nausea. The pain afterwards may subside in the part first affected, and acutely affect some distant organ, or a limb in the course of an artery. In this case, greater heat is often felt in the part where it exists. In addition, it may be remarked, that, though the symptoms of acute inflammation are present, it cannot be affirmed that any particular viscus is its seat, and the pains suddenly wander about the body, chiefly following the trunks of the larger arteries, whose pulsations become at intervals throbbing and violent. In the subsequent stages the pulse suddenly becomes weaker and slower, at other times very feeble and quick. Severe rigors are followed by profuse perspirations. The countenance is pale and extremely anxious, and the mental faculties are often affected. In the course of some particular artery a hardness, a

* "On the Spontaneous Obstruction of the Canals of the Larger Arteries.—Transactions of the Medico-Chirurgical Society of Edinburgh, vol. iii. part i. p. 105".

† "See Mr. Turner's Paper, (Op: Cit.) also Dublin Hospital Reports, vol. v. p. 17. Case of Arteritis in the Annual Reports of the Meath Hospital. By Dr. Graves and Dr. Stokes."

swelling, or a knotty feeling is discovered. The patient experiences the sensation '*that something has given way in the limb*.' The pulse is lost in one or more of the extremities, and the part becomes cold, perhaps paralyzed. But ere this period the *consequences* of inflammation have ensued, and the history refers to its second stage.

The symptoms are, however, unfortunately frequently much less prominent, and the disease declares itself only by its fatal consequences.

In the present case the paralysis of the left side of the body evidently depended on the abscess discovered in the right corpus striatum and thalamus, which first organ is most frequently found diseased in hemiplegia. The paralysis of the right leg, however, depended on the obstruction of the artery, and the imperfect supply of blood to the muscular and nervous tissues. The necessity of a larger supply of blood to muscle than is necessary for the purposes of nutrition has not, I think, sufficiently attracted the attention of physiologists in recent researches. It may, perhaps, be shewn that this supply has a direct relation to the *mechanical* phenomena developed in muscular action. This is not the place to insist on these opinions; but, whatever result be obtained by more extensive investigations, this case is illustrative of the paralysis depending on a *diminished supply of blood* to muscular fibre."

Case 2. This was a case of obliteration of the cava inferior, with fungus hæmatodes of the kidney.

The subject of the disease was not seen by the author till within a week of his decease. The patient had been a man of dissipated habits, and intemperate in the use of spirituous liquors. His faculties had been much injured by his disease, and no very clear history could be obtained. He had suffered from severe pain in the lumbar region, shooting in the course of the ureters, accompanied by sickness. There was no calculus in the bladder. He complained of severe head-aches, dizziness, insomnium, and occasional delirium. His legs and abdomen swelled, and his strength fled. It is needless to mention the remedies employed, as they all failed to produce any relief.

"On opening the cavity of the abdomen many quarts of serum were removed. A large tumour, treble the natural size of the kidney, was discovered growing from its texture. Externally it had an irregular lobulated form, and was of a deep red colour. On making an incision through the centre of this morbid growth into the structure of the kidney, it was discovered that it sprang from, and was intimately connected with its tissue, into which it graduated by insensible structural changes. Its consistence was spongy and soft, and its colour generally resembled that of muscle. Portions, however, of irregular form and various magnitude, and of a whitish or opaline colour, were scattered through it. Their texture was somewhat more fibrous, and they were bounded by defined lines, like membranous envelopes. Portions of the more vascular and redder mass were softened into a thick liquid of light red colour, and opaque, resembling a mixture of blood and pus; and other portions were passing, in various stages, to the same state. This morbid structure extended to the superior extremity of the kidney, graduating as below by insensible shades into the healthy texture of the viscus. An aperture was found near the origin of the pelvis of the kidney, connecting the tumour with the emulgent vein, which, as well as the Vena Cava, above the point where the emulgent vein opens into it, was filled with the softened matter above described. But from this point downwards till after its bifurcation, the cavity of the Vena Cava was entirely obliterated by the effusion of lymph, which was organized at the superior part, and passed gradually into coagulated blood in the portion near the origin of the iliac veins. Both the emulgent and the cava above the insertion of the latter were very considerably enlarged. A small quantity of the softened encephaloid tumour was found in the right auricle. The artery was of its natural size, and healthy, as were also the right kidney, and its vessels. In the liver were several tubercles of various magnitude, from that of a small bean to the size of a pigeon's egg. These, when cut into, were found to consist principally of white cerebriform matter, though interspersed, in some of the larger, with striæ of a fleshy colour like that of the diseased kidney. Some of these were manifestly approaching to the stage of softening, and from their centres a small quantity of a puro-sanguineous liquid exuded on pressure with the scalpel. In the superior lobe of the right lung was a small hard cartilaginous body of a greenish grey colour. The heart and its valves were healthy.

The circulation, in this case, appeared to have been chiefly carried on by the inosculations of the veins supplying the rectum and sigmoid flexure, with those which communicated with the intestines situated in the higher regions of the abdomen. Their enlargement was however not so great as might have been expected."

